# Systematics for types and effects of DNA variations

**DOI:** 10.1186/s12864-018-5262-0

**Published:** 2018-12-28

**Authors:** Mauno Vihinen

**Affiliations:** Department of Experimental Medical Science, Lund University, BMC B13, SE-22184 Lund, Sweden

**Keywords:** DNA variations, Mutation, Variation, Systematics, Variation ontology, VariO, Annotation, Databases, Ontology

## Abstract

**Background:**

Numerous different types of variations can occur in DNA and have diverse effects and consequences. The Variation Ontology (VariO) was developed for systematic descriptions of variations and their effects at DNA, RNA and protein levels.

**Results:**

VariO use and terms for DNA variations are described in depth. VariO provides systematic names for variation types and detailed descriptions for changes in DNA function, structure and properties. The principles of VariO are presented along with examples from published articles or databases, most often in relation to human diseases. VariO terms describe local DNA changes, chromosome number and structure variants, chromatin alterations, as well as genomic changes, whether of genetic or non-genetic origin.

**Conclusions:**

DNA variation systematics facilitates unambiguous descriptions of variations and their effects and further reuse and integration of data from different sources by both human and computers.

## Background

Variations at DNA are frequent and form the foundation of evolution. Some variants are related to diseases but many do not have any associated phenotype. The range of changes is very wide, from single nucleotide substitutions to changes in the number of entire chromosome sets. We can distinguish four categories, those in local DNA regions, such as genes; chromosomal variations; chromatin changes; and genome-wide alterations. To fully understand variants and their mechanisms and significance it is necessary to investigate them from different angles, e.g. to identify types of variants, but also to understand how they may affect structure, function, interactions, properties etc. For a systematic description of variations and their consequences, effects and mechanisms a framework called Variation Ontology (VariO) was developed [1]. As an ontology VariO facilitates systematic and detailed descriptions of variants. VariO includes terms for all kinds of alterations in DNA, RNA and protein.

Experimental studies provide the most reliable interpretation for variants and their effects and consequences. However, the huge volume of variants, e.g. about 3 million substitutions in a genome for a human individual, does not allow extensive experimental studies. Therefore, different kinds of prediction methods have been developed. The numbers of such tools are much higher for protein variants (see e.g. [2]). Non-coding variants are more difficult to predict largely due to lack of examples with known outcome. DeepSEA [3] is an example of a DNA predictor. For transcription factor binding sites and expression regulation, several approaches are available.

The Encyclopedia of DNA Elements (ENCODE) project has annotated functional elements at genomic regions, largely based on predictions [4]. There are data for transcription, transcription factor association, chromatin structure and histone modifications. For transcription factor binding sites and expression regulation, several predictors are available, reviewed in [5], that take into account sequence motifs, chromatin features and others. There are also methods to predict effects of *cis* regulatory elements and variants [6] including enhancers [7].

Dedicated methods are available for insertions and deletions whether affecting the reading frame or not [8–10]. When considering using these tools, one should bear in mind that most of them have not been systematically benchmarked as has been done for e.g. amino acid substitutions [11, 12]. Systematic method assessments are available for nucleosome position prediction methods [13, 14] as well as for predictors of topologically associating domains (TADs) [15].

Here, DNA variations, their types, functions, structural effects and properties are described in the systematic framework of VariO, similar to a previous article for protein variations [16]. As far as the author knows, this is the first systematic treatise of DNA variations and applicable to all organisms and kinds of variations and mechanisms. Variations at DNA level are important as such but also because they constitute the basis for inherited variations at RNA and protein levels. Examples are presented to highlight the different features of variants, usually in the context of human diseases.

### Databases for DNA variations

Numerous databases distribute DNA variation information. In Table 1 [17–49] examples of some widely used resources and types of databases are given. All the human genes or numerous genes are represented in general variation databases while locus specific databases (LSDBs) are more focused and are collected for individual genes/diseases or groups of them. Many LSDBs are considered as the most reliable sources for disease related variation data, along with ClinVar. Exome and complete genome databases contain complete variation datasets from several studies. As these data are sensitive due to being personal, access is limited, however they are available for research purposes. Ethnic and national databases typically contain details for several diseases in more focused groups. For variation frequency information in diverse populations, dedicated resources are available and can be used e.g. for variation interpretation when finding out likely benign alterations.

**Table 1:** Examples of DNA variation databases

Database	URL	Reference
General variation databases		
Ensembl Variation Database	http://www.ensembl.org/info/genome/variation/index.html	[17]
ClinVar	http://www.ncbi.nlm.nih.gov/clinvar/	[18]
Database of Short Genetic Variations (dbSNP)	http://www.ncbi.nlm.nih.gov/SNP/	[19]
Exome and complete genome sequences		
ExAC	http://exac.broadinstitute.org	[20]
NHLBI Exome Sequencing Project (ESP) Exome Variant Server (EVS)	http://evs.gs.washington.edu/EVS/	[21]
The 1000 Genomes Project	http://www.internationalgenome.org/	[22]
European Nucleotide Archive (ENA)	https://www.ebi.ac.uk/ena	[23]
Locus specific variation databases		
Leiden Open Variation Databases (LOVD)	http://www.lovd.nl/3.0/home	[24]
Universal Mutation Database (UMD)	http://www.umd.be/	[25]
ImmunoDeficiency Variation Databases (IDbases)	http://structure.bmc.lu.se/idbase	[26]
The TP53 web site	http://www.p53.fr/	[48]
Allele frequency databases		
The ALlele FREquency Database (ALFRED)	https://alfred.med.yale.edu/alfred/	[27]
FINDbase	http://www.findbase.org/	[28]
Allele Frequency Net Database (AFND)	http://www.allelefrequencies.net/	[29]
Allele Frequency Community (AFC)	http://www.allelefrequencycommunity.org/	[30]
Cancer variation databases		
Catalogue of Somatic Mutations in Cancer (COSMIC)	http://cancer.sanger.ac.uk/cosmic	[31]
The Cancer Genome Atlas (TCGA)	https://portal.gdc.cancer.gov/	[32]
International Cancer Genome Consortium (ICGC)	https://dcc.icgc.org/	[33]
Ethnic/national databases		
Pakistan Genetic Mutation Database	http://www.pakmutation.com/	[34]
The Singapore Human Mutation And Polymorphism Database	http://shmpd.bii.a-star.edu.sg/	[35]
Databases of genomic structural variations		
dbVar	https://www.ncbi.nlm.nih.gov/dbvar/content/human_hub/	[36]
Database of Genomic Variants (DGV)	http://dgv.tcag.ca/dgv/	[37]
Database of Genomic Variants archive (DGVa)	https://www.ebi.ac.uk/dgva	[38]
Mitelman Database of Chromosome Aberrations in Cancer	http://cgap.nci.nih.gov/Chromosomes/Mitelman	
Human Polymorphic Inversion Database (InvFEST)	http://invfestdb.uab.cat/	[39]
Repeat databases		
The European database of L1-HS retrotransposon insertions in humans (euL1db)	http://eul1db.unice.fr/	[40]
L1base, LINE-1 insertions	http://l1base.charite.de/l1base.php	[41]
SINEbase	http://sines.eimb.ru/	[42]
Short Tandem Repeat DNA Internet DataBase (STRBase)	https://strbase.nist.gov/	[43]
Methylation databases		
Methylation Bank (MethBank)	http://bigd.big.ac.cn/methbank	[49]
NGSmethDB	http://bioinfo2.ugr.es:8888/NGSmethDB/	[44]
miRNA target databases		
Polymorphism in microRNAs and their TargetSites (PolymiRTS)	http://compbio.uthsc.edu/miRSNP/	[45]
Somatic mutations altering microRNA-ceRNA interactions (SomamiR DB)	http://compbio.uthsc.edu/SomamiR/	[46]
DNA loop database		
R-loop DB	http://rloop.bii.a-star.edu.sg/	[47]

Databases have been established for many diseases, those for cancer contain large amounts of data. Structural variants form a special group of alterations, there are specific data collections for them. Several resources share information on short repeat sequences and of methylation. Dedicated databases list microRNA and target variants, as well as DNA loops.

## Variation ontology

For an efficient use, reuse, search and integration of variation information it is essential to describe it in a systematic way. VariO (http://variationontology.org/) was developed for the systematic description of variation types, effects, consequences and mechanisms [1]. The ontology is used to annotate information in databases at the three molecular levels: DNA, RNA and protein. Each of these levels contains further terms for *variation type*, *function*, *structure* and various *properties*. Here, DNA variation types and effects will be discussed. VariO annotations are always made in relation to a reference state, e.g. a reference sequence or a wild type property. A new version of VariO has been released with new terms, especially for DNA. VariO development continues, new terms are added and some rearrangements of already included terms are made when required, as in the latest releases for some areas in DNA and RNA terms. The basic structure of VariO has remained the same ever since first released, however new terms have been added, terms have been reorganized, clarified and redefined, when need has arisen. New terms, clarifications and updates can be suggested via the web site.

Systematic annotations consist of two parts: the VariO prefix and a number followed by the term. As an example, VariO:0132 is for “chromosomal variation”. The number with the prefix is mandatory for annotation, the term name can be derived with that information. This article is organized according to the VariO: DNA variations are divided into the four major sublevels - DNA variation type, function, structure and properties. Subheadings are VariO terms, in the text terms are written in quotation marks. Detailed guidelines for the use and annotation have been published [50]. Consistent database annotations can be made with the VariOtator annotation tool [51]. VariO annotations are already used in a number of databases including some of those in the LOVD (Leiden Open (source) Variation Database) LSDB system, such as BTKbase [52] and SH2base [53], as well as in UniProt [54] and VariBench [55]. VariO is available in several ways including the website, AmiVariO, Ontology Lookup Service (https://www.ebi.ac.uk/ols/ontologies/vario), OBO Foundry (http://www.obofoundry.org/ontology/vario.html), NCBO BioPortal (https://bioportal.bioontology.org/ontologies/VARIO), Ontobee (http://www.ontobee.org/ontology/VariO), AgroPortal (http://agroportal.lirmm.fr/ontologies/VARIO), FAIRsharing (https://fairsharing.org/bsg-s000776/) and others.

VariO is used to describe the outcome of the mutation, i.e. the changed nucleotides etc., not the mechanism that led to the alteration. The latter we cannot explain just by looking at the variant. Note that “mutation” (VariO:0139) in VariO means “any process generating variation”, not the outcome of these processes.

VariO annotations can be enriched with additional systematics, as described in the original article [1]. To provide details on the methods based on which the annotations are made, Evidence & Conclusion Ontology (ECO) terms [56] can be used to indicate whether and which laboratory experiments, computational methods, literature curation, or other means have been applied.

## DNA variation type (VariO:0129)

**Fig. 1:**
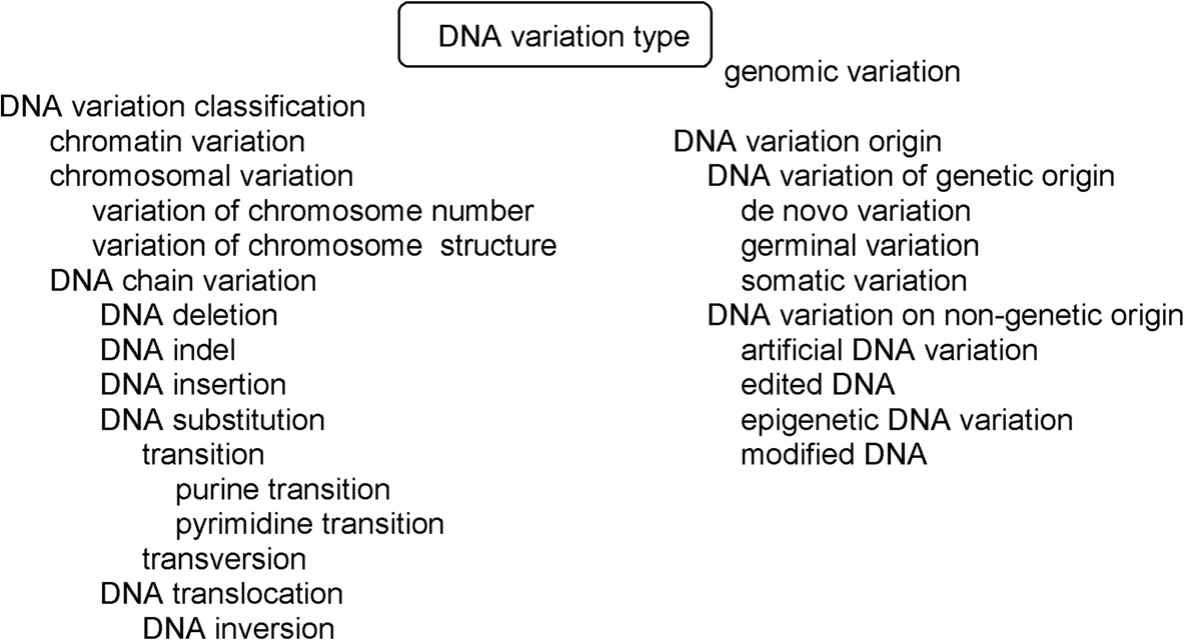
Terms to describe DNA variation types

Variation type in VariO provides a description of a variation in English (see Fig. 1). Variation type terms provide a brief description with commonly used terms. They are not intended to replace Human Genome Variation Society (HGVS) names [57] or the International System for human Cytogenetic Nomenclature (ISCN) [58], instead to provide an easily understandable description for human readers and computer applications. VariO terms can be used together with HGVS and ISCN nomenclature.

There are four levels for the descriptions: DNA chain, chromosomal, genomic and chromatin levels, depending on the type and size of the variation. With VariOtator, the variation type annotations at DNA, RNA and protein level can be made automatically, including for Leiden Open Variation databases (LOVD), from the HGVS names. In the following examples, HUGO Gene Nomenclature Committee (HGNC) names [59] are indicated for genes. The HGVS prefixes for DNA (c. for coding DNA, g. genomic sequence, m. mitochondrial) are used in the text. In some instances protein variants are discussed, they are indicated with prefix p.

### DNA variation classification (VariO:0322)

Histone variants or alterations in remodeler and modifier enzymes or their expression affect “chromatin variation” (VariO:0153). These alterations are frequent in cancers [60]. “Chromosomal variation” (VariO:0132) is either “variation of chromosome number” (VariO:0133) or “variation of chromosome structure” (VariO:0134). Down syndrome with trisomy of chromosome 21 [61] is an example of “variation of chromosome number”, while Rett syndrome due to an inversion in X chromosome [62] is a “variation of chromosome structure”.

**Fig. 2:**
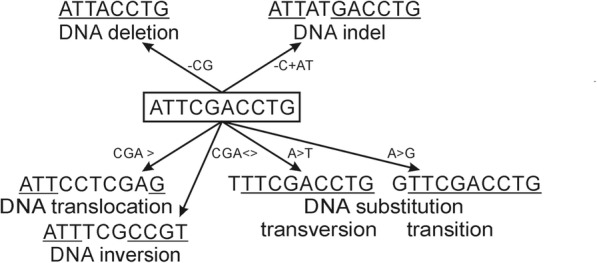
Examples of DNA chain variations. The original sequence is in the middle. In the variant sequences the original bases at original positions are underlined

Variations in the DNA chain occur e.g. within a gene or another functional unit, while chromosomal variations affect larger regions in chromosomes. The different types of DNA chain variations in a short sequence are shown in Fig. 2. Chromosomal variations are described in detail with “variation affecting DNA structure” (VariO:0155) annotations.

There are 5 categories of “DNA chain variation” (VariO:0135) types, some of them with subcategories. “DNA deletion” (VariO:0141) of G from region for intron 3 (g.101374535del) in *BTK* gene coding for Bruton tyrosine kinase causes a splice defect and leads to X-linked agammaglogulinemia (XLA) [63]. “DNA indel” (VariO:0143) is a variant that is due to both insertion and deletion. Alteration from C to TG in *BTK* gene coding for exon 17 (c.1684_1685delinsT) causes XLA due to RNA frameshift and truncated protein [64] is an example of a DNA indel. The original base C is deleted and TG inserted instead. “DNA insertion” (VariO:0142) introduces a new base(s) to the DNA, such as insertion of T to *BTK* gene for exon 3 (g.101374623insT) introducing a new stop codon [65]. “DNA substitution” (VariO:0136) is the most common single nucleotide variation type and DNA variation in general. G to C substitution in the *BTK* gene coding for the TH domain (g.101362620C > G) causes amino acid substitution in Zn finger leading to XLA [66]. DNA substitutions are either transitions or transversions. “Transition” (VariO:0313) changes a purine base (A, G) to another purine or a pyrimidine (C, T) to another pyrimidine. “Transversion” (VariO:0316) is a substitution from a purine to pyrimidine or vice versa. The G to C substitution is a transversion. Transitions can be classified further to “purine transition” (VariO:0315) and “pyrimidine transition” (VariO:0314) .

When a sequence stretch is moved to a new location within a chromosome it is called for “DNA translocation” (VariO:0144). “DNA inversion” (VariO:0145) is a special type of translocation where the sequence is inverted to its original place. Microinversions are rare, such as a 95 nucleotide inversion at 22q11.21 (Database of Genomic Variants nsv1129408) [67].

Genomic variations affect the entire genome. Autopolyploidy, which means duplication of chromosome sets originating from the same organism, is an example of “genomic variation” (VariO:0131) and common in human liver [68].

### DNA variation origin (VariO:0127)

There are two types of “DNA variation origin” (VariO:0127), namely “DNA variation of genetic origin” (VariO:0130) and “DNA variation of non-genetic origin” (VariO:0146). Variants of genetic origin have appeared on DNA (or RNA) level and therefore directly affect the protein, when in a coding region.

Insertion in the non-coding region of exon 2 in *BTK* is a “de novo variation” (VariO:0444) and has occurred in that invididual [69], while G to C substitution (c.1685G > C) for codon 562 causing p.R562P substitution in protein is a “germinal variation” (VariO:0445) [70] that has occurred in the germ cell of the mother. Melanoma-related A to T transversion in *GNA11* (G protein subunit alpha 11) gene leading to a G209 L substitution is a “somatic variation” (VariO:0446) [71].

Several variation types are of non-genetic origin. Replacement of A by C in *BTK* leading to the amino acid substitution p.Y334S was made in a construction and is thus an “artificial DNA variation” (VariO:0172) [72]. Novel genome editing technologies allow generation of specific DNA alterations e.g. to correct for genetic defects as in β-thalassemia [73] leading to “edited DNA” (VariO:0407). This example is an artificial variation, but genomic editing appears naturally in some organisms. Changes in DNA methylation pattern are a form of “epigenetic DNA variation” (VariO:0147) and are associated to systemic lupus erythematosus due to changes in transcription activation [74]. DNA lesion, such as incorporation of 8-hydroxyguanine to DNA, causes a form of “modified DNA” (VariO:0337) [75].

## Variation affecting DNA function (VariO:0148)

DNA molecules have several functions. Some DNA molecules have catalytic deoxyribozyme activities. Self-catalyzed sequence-specific DNA depurination is the only known DNA catalytic activity [76]. Variations to the required cruciform structure could have an “effect on catalytic DNA activity” (VariO:0412).

Deletion of G from the region for intron 3 in *BTK* gene causes splice defect and XLA [63] due to “effect on DNA information transfer” (VariO:0150). The type of DNA variation affects DNA repair mechanisms. T/G or U/G mismatches are corrected by base excision repair, but lead also to increased frequency of variations i.e. have an “effect on DNA repair” (VariO:0151) as reviewed in [77]. Variation A to C in the TATA box of the *HBB* gene for hemoglobin subunit beta leads to β-thalassemia [78] because of “effect on regulatory function of DNA” (VariO:0152). DNA replication fidelity can be affected by numerous factors including DNA variations such as DNA adducts caused by reactions with e.g. environmental mutagens, and sequence context [79, 80], thus having an “effect on DNA replication” (VariO:0154).

Variations at two major *TERT* (telomerase reverse transcriptase) gene promoter sites are frequent in melanoma patients and generate binding sites for Ets/TCF transcription factors [81]. These variants are classified to have “effect on transcription” (VariO:0149).

## Variation affecting DNA property (VariO:0227)

DNA properties affected by variations are described by terms in this category. Insertion of T to the *BTK* gene coding for exon 3 introduces a new stop codon [65] and has “association of DNA variation to pathogenicity” (VariO:0229). Variation c.82C > T in *BTK* causing p.R28C [82] affects “conservation of DNA variation site” (VariO:0231) [83] by affecting highly conserved position. Variations at *TERT* gene promoter in melanoma patients generate binding sites for Ets/TCF transcription factors [81] and have “effect on DNA interaction” (VariO:0230).

## Variation affecting DNA structure (VariO:0155)

**Fig. 3:**
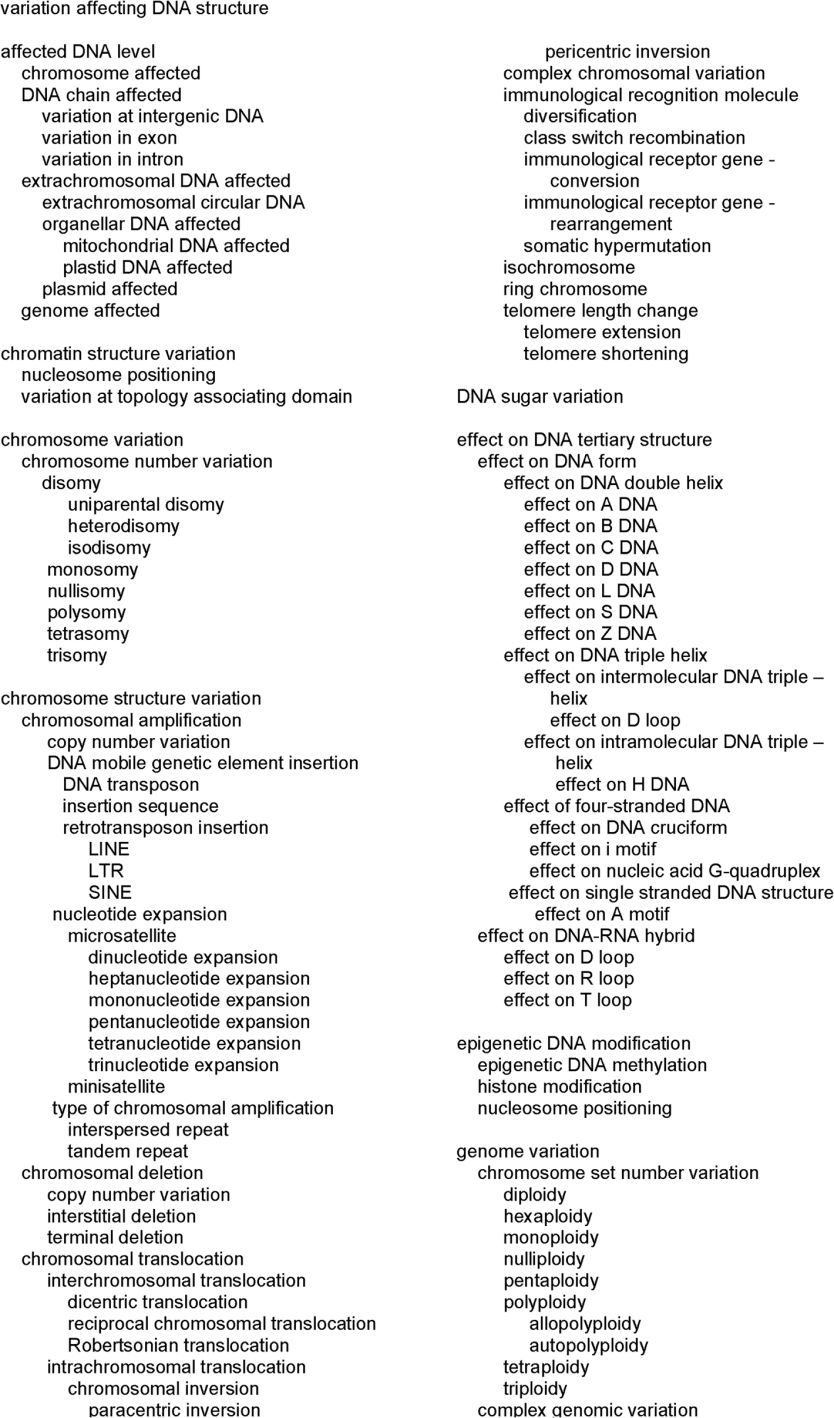
Terms for describing variations affecting DNA structure

DNA structure and architecture have several levels and layers. In addition to the double stranded form there are single and multiple stranded DNA forms and with and without RNA. Depending on the cell cycle stage, the DNA chain condensation varies greatly from a very tightly packed form to an elongated DNA chain. The entire structure of this most extended part of DNA terms is depicted in Fig. 3.

### Affected DNA level (VariO:0159)

DNA level terms are used to indicate what kind of DNA molecule and region is affected by the variation. A Rett syndrome-causing inversion in the X chromosome [62] has “chromosome affected” (VariO:0164). “DNA chain affected” (VariO:0160) has three subcategories. *TERT* gene promoter variants in melanoma patients that generate binding sites for Ets/TCF transcription factors [81] are “variation at intergenic DNA” (VariO:0163). G to C substitution in the *BTK* gene leads to amino acid substitution at zinc finger motif causing XLA [66] and is a “variation in exon” (VariO:0162). Deletion of G from the region for intron 3 in the *BTK* gene causes splice defect and XLA [63] and is a “variation in intron” (VariO:0161) .

G to A substitution coding for codon 467 (p.A467T) in the mitochondrial *POLG* (DNA polymerase gamma, catalytic subunit) gene causing progressive external opthalmoplegia and other diseases [84] has “extrachromosomal DNA affected” (VariO:0072) of type “organellar DNA affected” (VariO:0448) and even more specifically “mitochondrial DNA affected” (VariO:0450). Mitochondria are essential organelles for energy production in eukaryotes, whereas the other compartments with their own DNA, plastids are unique for plants and algae and appear only in some eukaryotes. Substitutions in the plastid *infA* (IF1 homolog) gene in spring barley lead to cytoplasmic line 2 (CL2) syndrome [85] and have “plastid DNA affected” (VariO:0451).

There are two additional forms of “extrachromosomal DNA affected” (VariO:0072). Variants to a H group plasmid change its maintenance as temperature sensitive in *Escherichia coli* [86]. In this case the variant has “plasmid affected” (VariO:0391). Plasmids are independently replicating circular DNA units common in bacteria but can appear also in other organisms. Plasmids can be transferred between cells, even organisms. Many plasmids contain toxin or antibiotic resistance genes. “Extrachromosomal circular DNA” (VariO:0449) is common in many organisms and are widely variable in size and contents as they originate from material in linear chromosomes [87].

Trisomy of chromosome 21 [61] has “genome affected” (VariO:0391).

### Chromatin structure variation (VariO:0226)

GAA triplet expansions in the *FXN* (frataxin) gene are the most usual cause of Friedreich ataxia, a form of progressive damage of the nervous system. The triplet expansion alters nucleosome positioning so that transcriptional activity is reduced because the start site is not accessible [88] being a “chromatin structure variation” (VariO:0226) due to effect on “nucleosome positioning” (VariO:0158).

Topologically associating domains (TADs) are a higher order chromatin structures where genomic regions interact with each other. These regions are thought to be involved e.g. in regulation. “Variation in topology associating domain” (VariO:0454) appears in diseases including various forms of cancers where boundaries of TADs are altered [89].

### Chromosome variation (VariO:0176)

“Chromosome variation” (VariO:0176) is divided into two categories “chromosome number variation” (VariO:0206) and “chromosome structure variation” (VariO:0180).

#### Chromosome number variation (VariO:0206)

**Fig. 4:**
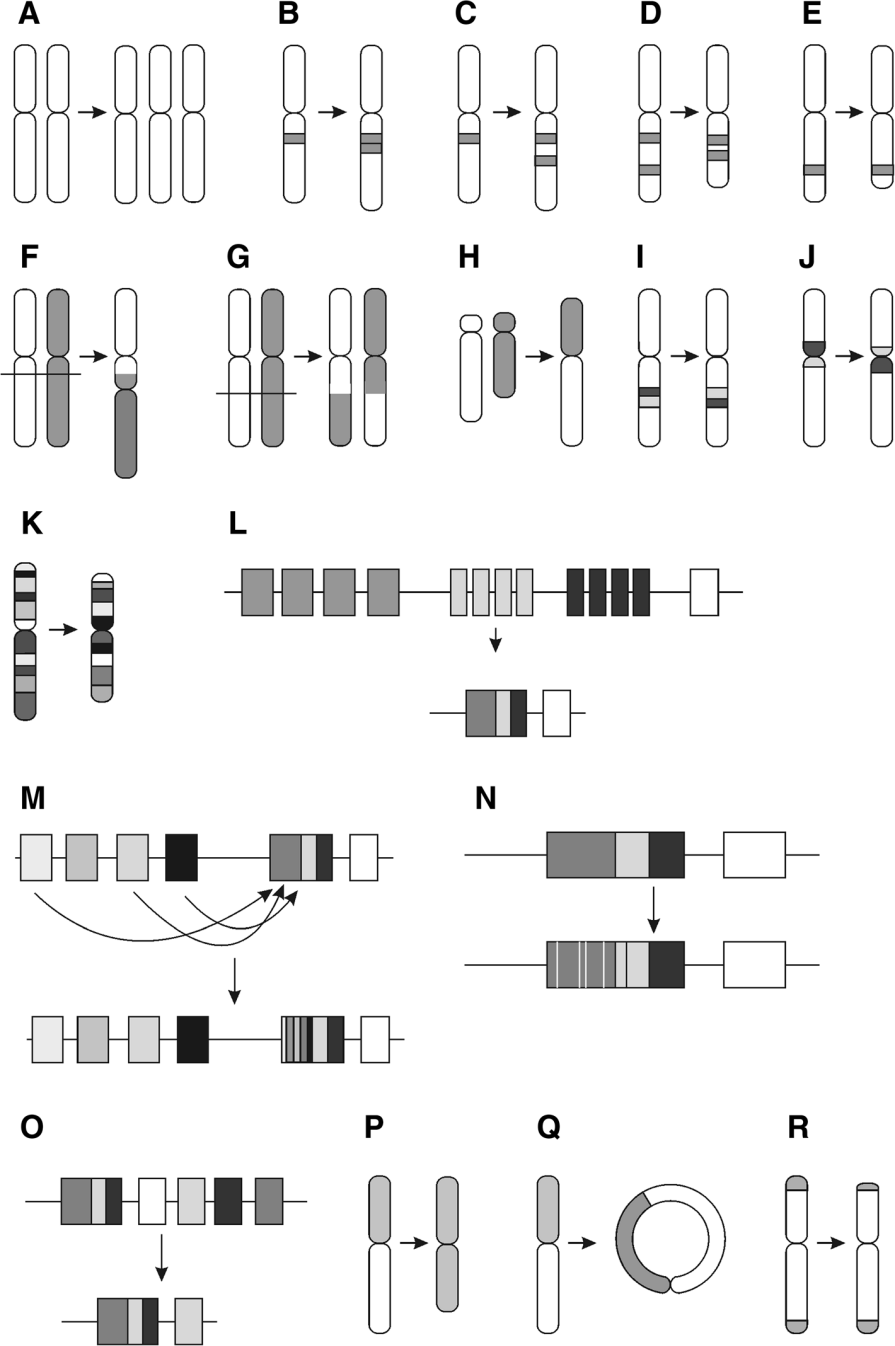
Visualization of chromosomal structure variations. **a** Chromosome number variation, trisomy as an example. **b** Chromosomal amplification, more specifically copy number variation of type tandem repeat. **c** Intrespersed repeat chromosomal amplification. **d** Interstitial chromosomal deletion, (**e**) terminal deletion. There are several forms of chromosomal translocations. These include, (**f**) dicentric translocation, which is a form of interchromosomal translocation, (**g**) reciprocal translocation, **h** Robertsonian translocation, (**i**) paracentric inversion which is also intrachromosomal translocation, and (**j**) pericentric inversion. **k** Complex chromosomal variation. Several chromosomal changes are involved in immunological recognition molecule diversification, including (**l**) immunological receptor gene rearrangement, (**m**) immunological receptor gene conversion, (**n**) somatic hypermutation, and (**o**) class switch recombination. The gene segments are from the left V, D, J and C. There are up to tens of fragments in each segment type. **p** Isochromosome, (**q**) ring chromosome. **r** Telomere length variations, specifically telomere shortening. Note that the sizes of the telomeres in the ends of chromosomes as well as the telomere shortening are exaggerated

Variations in this category are used to describe changes in the number of chromosomes. “Nullisomy” (VariO:0212), lack of both chromosomal pairs, is not viable in human. “Disomy” (VariO:0208) is the normal genetic setting e.g. in human. Prader-Willi syndrome is caused by a lack of expression of genes in paternal chromosome in a segment of chromosome 15. There are three mechanisms behind the condition, one of them is “uniparental disomy” (VariO:0209) [90]. “Heterodisomy” (VariO:0211) appears when the non-identical chromosomes are from one parent. Paternal heterodisomy in chromosome 1 involving the *LYST* (lysosomal trafficking regulator) gene containing a substitution introducing a stop codon on RNA causes Chediak-Higashi syndrome [91]. In “isodisomy” (VariO:0210) there is a duplication of a single chromosome from one parent. Paternal genome-wide “uniparerental disomy” (VariO:0209), a condition where both copies of a chromosome or its part in a diploid cell or organism are from just one parent, in a patient causes Beckwith-Wiedemann syndrome [92]. Down syndrome is caused because of complete or partial “trisomy” (VariO:0207) of chromosome 21 [61] (Fig. 4a). In “tetrasomy (VariO:0213)” there are four copies of the chromosome and in “polysomy” (VariO:0303) more than the normal number.

#### Chromosome structure variation (VariO:0180)

The numerous types of variations in this category are depicted in Fig. 4.

##### Chromosomal amplification (VariO:0183)

Numerous variation types and mechanisms affect the number of chromosomal region copies. “Copy number variation” (CNV) (VariO:0187) ranges in size from 1 kb up to several megabases and can be either amplification or deletion (Fig. 4b). CNV duplication of *LAMB1* (laminin B1) gene causes autosomal dominant leukodystrophy [93].

##### DNA mobile genetic element insertion (VariO:0192)

“DNA mobile genetic element insertion” (VariO:0192) and its subcategories are used to describe insertions of various mobile genetic elements. The transposition of a “DNA transposon” (VariO:0378) is catalysed by transposase enzymes with a cut-and-paste mechanism [94].

“Insertion sequence” (IS) (VariO:0392) is a short transposable element that contains only genes for transposition activity. Thereby, IS differs from other transposons that can contain or can be loaded with additional genetic material. Insertion sequence 2404 specific for *Mycobacterium ulcerans* originating from a crayfish can cause Buruli ulcer, a severe skin infectious disease in human [95].

“Retrotransposon insertion” (VariO:0377) means a transposon insertion via RNA intermediate which is reverse transcribed to DNA. There are three types of retrotransposons: LINE, LTR and SINE. “LINE” (VariO:0379), long interspersed element, copies constitute totally about 17% of the human genome [96]. Insertion of LINE elements of about 6000 bp long to or close to human genes leads to a number of diseases including familiar hypocalciuric hypercalcinemia and neonatal severe hyperparathyroidism [97]. “SINE” (VariO:0380), short interspersed nuclear element, is 100–700 nucleotides long and requires LINE for replication. Alu element is the most common form of SINE and involved in numerous human diseases [98]. “LTR” (VariO:0388) (long terminal repeat) transposons form the third category of retrotransposons. They are in size between 100 and 5000 bp. Similar to SINEs, LTRs require LINE for transposition.

“Nucleotide expansion” (VariO:0430) is a large group of variations where repeated nucleotide sequences are inserted to DNA. “Microsatellite” (VariO:0188) means repetitive sequences formed by units of one to six nucleotides. CAG expansion in the *HTT* gene for huntingtin is an example of “trinucleotide expansion” (VariO:0189) [99]. This microsatellite expansion introduces polyglutamine tract of variable length to the amino terminus of the encoded protein. There are terms from “mononucleotide expansion” (VariO:0190) to “heptanucleotide expansion” (VariO:0452) to describe these types of variants.

“Minisatellite” (VariO:0186) is a somewhat longer repeated sequence unit, in length from 10 to 60 bp, repeated up to 50 times. 48 bp minisatellite in dopamine receptor D4 gene, *DRD4*, is associated with Tourette syndrome, a neuropsychiatric disease [100].

“Type of chromosomal amplification” (VariO:0427) indicates whether the amplification is interspersed (Fig. 4c) or tandem repeat (Fig. 4b). Insertion of Alu element, a LINE transposon, is an example of “interspersed repeat” (VariO:0184), where the repeat units are separated from each other [97]. CAG trinucleotide repeat in Huntington’s disease is a form of “tandem repeat” (VariO:0185) [99].

##### Chromosomal deletion (VariO:0193)

Variants with “chromosomal deletion” (VariO:0193) are highly variable in size. “Copy number variation” (VariO:0187) can in addition to increasing copies of a DNA stretch also mean deletion. Williams-Beuren syndrome-causing deletions at 7q11.23 appear in the middle of the chromosome 7 [101] and are thus of “interstitial deletion” (VariO:0194) type (Fig. 4d). Deletions at chromosome 11 leading to Jacobsen syndrome are 5 to 20 Mb long and typically include the chromosome end [102] and are thus “terminal deletion” (VariO:0195) (Fig. 4e).

##### Chromosomal translocation (VariO:0197)

“Chromosomal translocation” (VariO:0197) rearranges genomic regions by moving them within and between chromosomes. There are several types of these changes as depicted in Fig. 4. When translocation occurs between coding regions gene fusions occur like the Philadelphia chromosome in *BCR-ABL1* fusion between chromosomes 9 and 22 [103], which is a hallmark of chronic myelogenous leukemia. “Interchromosomal translocation” (VariO:0202) occurs between different chromosomes, e.g. t(11;14)(q13;q32) in mantle cell lymphoma patients [104]. In “dicentric translocation” (VariO:0405) both the joined segments contain a centromere (Fig. 4f). The acentric segments are lost. This kind of variation leads e.g. to Kabuki syndrome [105]. “Reciprocal chromosomal translocation” (VariO:0203) happens between two chromosomes, such as t(11;14)(q13;q32) in mantle cell lymphoma patients [104] (Fig. 4g). “Robertsonian translocation” (VariO:0204) is a special type of translocation where the long arms of chromosomes are fused (Fig. 4h). This occurs between so called acrocentric chromosomes, which have very short p arms. In human, chromosomes 13, 14, 15, 21, 22 and Y are acrocentric. Infertile population has 10% increased prevalence of Robertsonian translocations compared to general population (1% vs 0.1%). Translocation rob(14;15)(q10:q10) is one such variation among females with recurrent abortions [106].

“Intrachromosomal translocation” (VariO:0198) occurs within one chromosome. “Chromosomal inversion” (VariO:0199) is a special type of translocation where the segment is joined inverted end to end back to the same chromosome (Fig. 4i). “Paracentric inversion” (VariO:0200) occurs within a single chromosome arm, such as in the X-chromosome in Rett syndrome patient where the epigenetic changes lead to overexpression of *MECP2* (methyl-CpG binding protein 2) gene [62] (Fig. 4i). “Pericentric inversion” (VariO:0201) includes the centromere, as an example leading to disruption of the *NSD1* (nuclear receptor binding SET domain protein 1) gene in Sotos syndrome [107] (Fig. 4j).

##### Complex chromosomal variation (VariO:0196)

“Complex chromosomal variation” (VariO:0196) leads typically to a complex phenotype, as in the patient with myeloid leukemia associated with Down syndrome [108] (Fig. 4k).

##### Immunological recognition molecule diversification (VariO:0447)

To achieve the huge amount of variability to immunological recognition molecules (antibodies, B and T-cell receptors, and major histocompatibility complex type I and II) special mechanisms have evolved. The human body can generate up to 10 billion different antibodies, thus effective diversity generating mechanisms are required as there are only about 22,000 genes in man.

“Immunological receptor gene rearrangement” (VariO:0166) is the major somatic recombination step where fragments for immunological receptor genes are joined to form a gene [109] (Fig. 4l). During “immunological receptor gene conversion” (VariO:0170) secondary diversification happens by replacing homologous DNA segments [110] (Fig. 4m). During “somatic hypermutation” (VariO:0168) variations are introduced to the antigen variable region [111] (Fig. 4n).

“Class switch recombination” (VariO:0169) is the final diversification step for antibodies where immunoglobulin M is switched to other isotypes by changing a portion of the heavy chain coding region (see [111]) (Fig. 4o).

##### Isochromosome (VariO:0181)

Isochromosome has one arm duplicated and the other one completely lacking (Fig. 4p). An example is the tetrasomy 18p syndrome where the isochromosome appears in addition to the normal chromosome pair [112].

##### Ring chromosome (VariO:0182)

“Ring chromosome” has its ends joined to form a ring structure (Fig. 4q). In ring chromosome 20 syndrome patients have refractory epilepsy and other symptoms [113].

##### Telomere length change (VariO:0177)

Telomeres are repetitive structures in the chromosome ends which are required for chromosome replication. During this process they are shortened because Okazaki fragments acting as RNA primers prevent complete replication. “Telomere extension” (VariO:0179) means variation that extends telomere [114]. In “telomere shortening” (VariO:0178) the telomere structure is shortened, a phenomenon that is related to many diseases (see [115]) (Fig. 4r).

### DNA sugar variation (VariO:0434)

DNA stands for deoxyribonucleic acids. It is composed of nucleotides, deoxyribose sugars, and phosphate groups. Most DNA variations affect nucleotides, however, “DNA sugar variation” (VariO:0434) does also exist e.g. due to carcinogens [116] and have special properties that could be beneficial for biotechnological and research applications [117].

### Effect on DNA tertiary structure (VariO:0171)

DNA tertiary structure means the three-dimensional shape of the DNA. Primary structure indicates the nucleotide sequence, secondary structure the base pairing of the molecule, and quarternary structure describes intermolecular interactions or interactions with other molecules. These structural levels are analogous to protein structural levels. Experimentally determined DNA structural forms are available at ProteinData Bank (PDB) [118] and Nucleic Acid Database (NDB) [119]. The structures were visualized with Jmol: an open-source Java viewer for chemical structures in 3D (http://jmol.sourceforge.net/).

#### Effect on DNA form (VariO:0167)

“Effect on A-motif” (VariO:0455) is an example of “effect on DNA form” (VariO:0167), more defined as “effect on single stranded DNA structure” (VariO:0455). A-motif has a single-stranded helical structure at alkaline and neutral pH while at acidic pH it forms a right-handed helical duplex. The structure requires A-rich DNA or RNA sequence and is important e.g. for the mRNA molecules that contain long poly-A tails.

##### Effect on DNA double helix (VariO:0390)

**Fig. 5:**
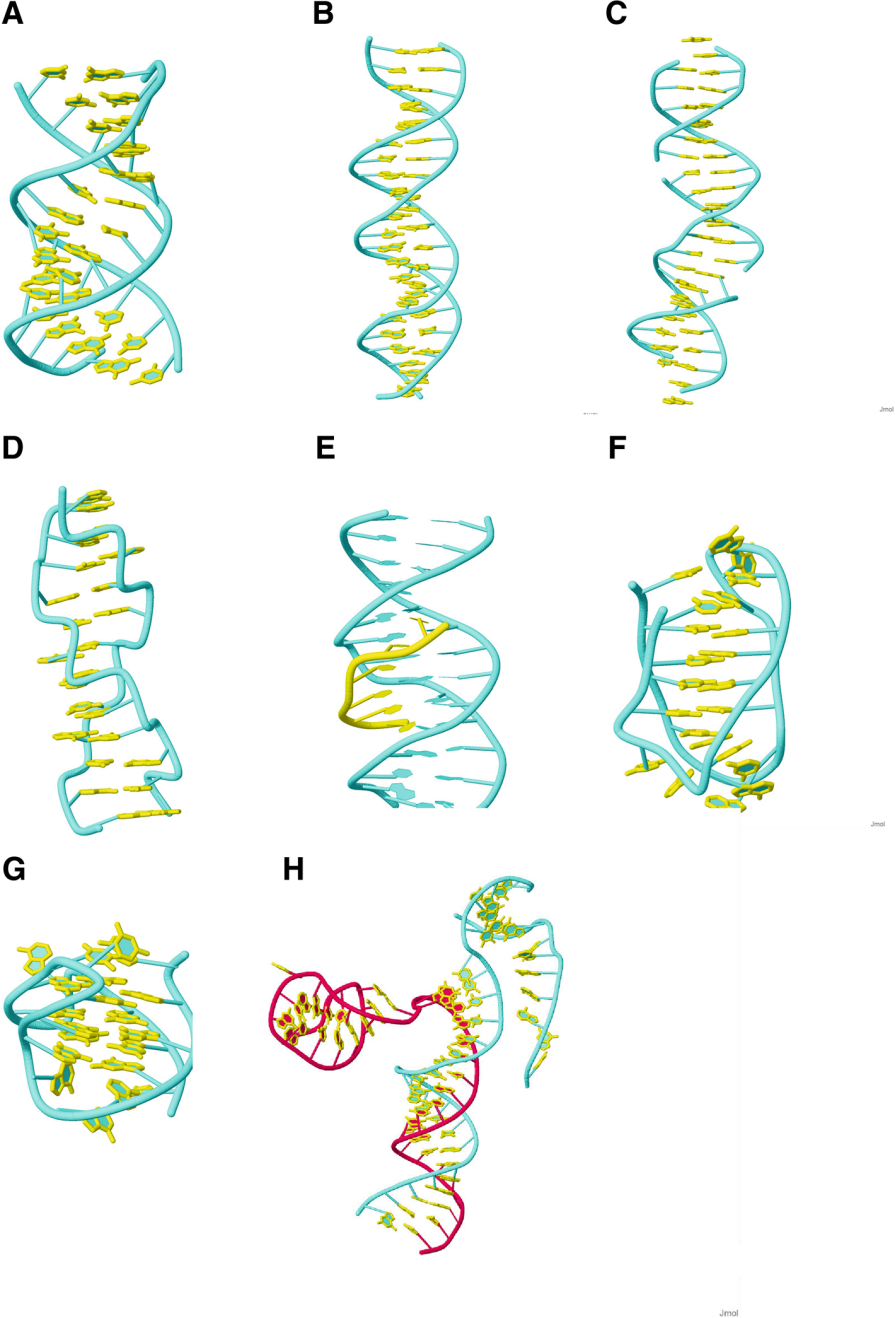
Three dimensional structures of DNA forms. Double helical structures. **a** A DNA (5iyg) [120], **b** B DNA (5f9i), **c** D DNA (5vy6) [121], and (**d**) Z DNA (4ocb) [122]. **e** Triple helix structure (1bwg) [125]. Four-stranded DNA structures (**f**) i motif (PDB entry 1el2) [130], and (**g**) G-quadruplex (2kzd) [142]. **h** DNA-RNA hybrid structure of type R loop (5mga) [137]. The DNA backbone is shown in cyan and the nucleotide bases with yellow. In H, the RNA chain backbone is in red

Most common of the DNA double helix structures is the B-form, however, there are numerous others. They have different conformations, such as A DNA [120], D DNA [121] and Z DNA [122] (see Figs. 5a to d), defined by the geometry of the DNA helices including e.g. the helix direction, rotation and number of base pairs per turn, inclination axis, rise and pitch/turn ratio etc. These molecules are right-handed except for Z-DNA which has a more open helix structure, which can be formed by alternating purine-pyrimidine sequences (Fig. 5d). These stretches can lead to the formation of deletions [123] and have an “effect on DNA double helix” (VariO:0390), more specifically “effect on Z DNA” (VariO:0421). Non B-DNA forms are involved in a number of diseases, see e.g. [124].

##### Effect on DNA triple helix (VariO:0175)

“Effect on DNA triple helix” (VariO:0175) means alteration to triple helical nucleotide chain structure. “Effect on D loop” (VariO:0433) is a form of “effect on intermolecular DNA triple helix” (VariO:0423). In this structure the strands in double-stranded DNA are separated and one of them pairs with a third strand which can be DNA or RNA (Fig. 5e) [125]. D loops are essential for the replication of mitochondrial DNA, which is circular. Variants at the D loop are common in cancers [126] and in some other diseases.

“Effect on intramolecular DNA triple helix” (VariO:0422) is the other type. The triple helix in H DNA requires mirror repeat symmetry. Supercoiling provides energy for opening of double-stranded DNA, then one of the chains swivels its background parallel to the remaining duplex DNA to form a triple helical structure. These are abundant in genomes and appear e.g. on regions that regulate expression of many genes involved in diseases. Variation can affect these structures and have “effect on H DNA” (VariO:0419) [127].

##### Effect on four-stranded DNA (VariO:0420)

“Effect on four-stranded DNA” (VariO:0420) means change to DNA structures where four chains are involved. DNA cruciform is formed on inverted repeat sequences when they form a cross-shaped structure with intrastrand base pairing. There are two conformations, in extended conformation the arms are at tips of a tetrahedron, whereas in closed conformation the arms are almost parallel. Cruciforms are involved in numerous interactions at DNA usage processes including gene expression regulation, replication and recombination [128]. Variations can have “effect on DNA cruciform” (VariO:0394). Cruciform structures are prone for translocations and DNA instability [129].

i-Motifs appear in C-rich sequences. Two parallel C-rich strands that form a duplex are intercalated in antiparallel orientation, see Fig. 5f [130]. The structures are uni-, bi-, or tetramolecular. Variations at these C-rich segments can have an “effect on i-motif” (VariO:0174). The *MYC* (MYC proto-oncogene, bHLH transcription factor) gene has in its promoter region seven nuclease sensitive element (NHE) III_1_ regions. Its expression is mainly (up to 90%) regulated by NHE III_1_ which can form an i-motif structure [131].

“Effect on nucleic acid G-quadruplex” (VariO:0173) describes changes where a G-quadruplex structure is involved [132] (Fig. 5g). These structures can be unimolecular, bimolecular or tetramolecular, and the chains in the two first ones can be either parallel or antiparallel, and formed by DNA, RNA or DNA-RNA hybrids [133]. Certain diseases are associated to these structures, including neurological diseases such as fragile X syndrome [134].

##### Effect on DNA-RNA hybrid (VariO:0424)

DNA and RNA chains can bind complementarily and form hybrids. D loop is one such structure.

R loop consists of a DNA:RNA hybrid and a displaced single-stranded DNA. The RNA strand is produced by transcription. These loops are rather rare and instable, being targets for nuclease cleavage [135]. They are implicated in human diseases, such are trinucleotide repeat-associated diseases [136]. Changes to these hybrids can have an “effect on R loop” (VariO:0431) [137] (Fig. 5h). R-loop DB [47] includes both predicted and detected R loops in 8 organisms, including human.

T loops appear on telomeres where the single stranded chromosome terminus forms a loop to protect the DNA repair system from recognizing them [138]. T loop is part of a large complex in which several proteins are involved, in human the sheltering complex of six proteins. Variations to these structures cause “effect on T loop” (VariO:0432) [138].

### Epigenetic DNA modification (VariO:0156)

Epigenetic changes are heritable traits that do not change the DNA sequence. There are three major types of “epigenetic DNA modification” (VariO:0156), including DNA methylation, histone modification and nucleosome positioning.

“Epigenetic DNA methylation” (VariO:0157) occurs almost exclusively on cytosines at CpG dinucleotides in C + G rich regions called CpG islands. Methylations in these islands are often associated to gene silencing including genomic imprinting, which causes monoallelic gene expression. DNA methylation is significantly affected in systemic lupus erythematosus including numerous cytokine genes. An example of “epigenetic DNA methylation” (VariO:0157) is decreased methylation of the interleukin 1 receptor type 2 gene, *IL1R2*, which is a suppressor for IL1 signalling that leads to downregulation of IL1 and can be used as a biomarker for lupus [139]. Further, trimethylation of histone H3 at lysine 4 (H3K4) molecules at *PTPN22* (protein tyrosine phosphatase, non-receptor type 22) and *LRP1B* (LDL receptor related protein 1B) genes positively correlate with lupus severity and is annotated as “histone modification” (VariO:0453).

The GAA triplet expansion of the *FXN* gene in Friedreich ataxia alters nucleosome positioning and reduces transcription by making the start site not accessible [88]. This is an example of “nucleosome positioning” (VariO:0158).

### Genome variation (VariO:0428)

Genome-wide alterations are described at this level.

“Chromosome set number variation” (VariO:0215) is used to annotate variations that affect the entire chromosome set number. The variations range from “nulliploidy” (VariO:0221) to polyploidy (VariO:0218), from 0 to several genomic copies, respectively. “Polyploidy” (VariO:0218) appears naturally also in some human cells including liver [68]. In “allopolyploidy” (VariO:0220) the chromosome sets originate from different organisms and is quite common in plants, such as in wheat [140]. In “autopolyploidy” (VariO:0219) the chromosome sets originate from the same organism, as in the human liver polyploidy [68].

“Complex genomic variation” (VariO:0429) describes genomic variations that contains several complex components within a single chromosome or between several ones. In chromothripsis a chromosome or several is shattered into segments some of which are randomly combined [141] and other segments are lost. This is an ultimate example of “complex genomic variation” (VariO:0429).

## Conclusions

VariO facilitates a detailed description of all kinds of DNA variants and their effects and consequences. These annotations can be made for any organism. DNA has four major sublevels for terms: variation type, function, structure, properties. DNA molecules have four levels: DNA chain, chromosome, chromatin and genome. By combining the terms, very detailed annotations are possible. By applying Evidence & Conclusion Ontology annotations [56] the quality and type of methods used or obtaining the data for the annotations can be described. For consistent annotation, the use of VariOtator tool [51] is recommended. It can generate variation type annotations automatically from HGVS descriptions and be directly ported to LOVD databases. Other types of annotations are made manually, VariOtator writes the annotation summary once all terms for a variant have been selected. VariO annotations will make data integration easier and more reliable. In this article, the full spectrum of DNA variations and their effects are presented in a systematic way with examples.

## Abbreviations

BTK: Bruton tyrosine kinase
CNV: Copy number variation
DRD4: Dopamine receptor D4
ECO: Evidence & Conclusion Ontology
ENCODE: The Encyclopedia of DNA Elements
FXN: Frataxin
GNA11: G protein subunit alpha 11
H3K: Histone H3 at lysine 4
HBB: Hemoglobin B
HGNC: HUGO Gene Nomenclature Committee
HGVS: Human Genome Variation Society
HTT: Huntingtin
IL1R2: Interleukin 1 receptor type 2
infA: IF1 homolog
IS: Insertion sequence
ISCN: International System for human Cytogenetic Nomenclature
LAMB1: Laminin subunit beta 1
LINE: Long interspersed element
LOVD: Leiden Open (source) Variation Database
LRP1B: LDL receptor related protein 1B
LSDB: Locus specific variation database
LTR: Long terminal repeat
LYST: Lysosomal trafficking regulator
MECP2: Methyl-CpG binding protein 2
MYC: MYC proto-oncogene, bHLH transcription factor
NDB: Nucleic Acid Database
NHE: Nuclease sensitive element
NSD1: Nuclear receptor binding SET domain protein 1
PDB: Protein Data Bank
POLG: DNA polymerase gamma, catalytic subunit
PTPN22: Protein tyrosine phosphatase, non-receptor type 22
SINE: Short interspersed nuclear element
TAD: Topologically associating domain
TERT: Telomerase reverse transcriptase
VariO: Variation Ontology
XLA: X-linked agammagolubulinemia
